# Transcriptome profiles reveal gene regulation of peanut (*Arachis hypogaea* L.) nodulation

**DOI:** 10.1038/srep40066

**Published:** 2017-01-06

**Authors:** Ze Peng, Fengxia Liu, Liping Wang, Hai Zhou, Dev Paudel, Lubin Tan, James Maku, Maria Gallo, Jianping Wang

**Affiliations:** 1 Agronomy Department, University of Florida, Gainesville, FL 32610, USA; 2 State Key Laboratory of Plant Physiology and Biochemistry, National Center for Evaluation of Agricultural Wild Plants (Rice), China Agricultural University, Beijing 100193, China; 3 State Key Laboratory for Conservation and Utilization of Subtropical Agro-bioresources, Key Laboratory of Plant Functional Genomics and Biotechnology of Guangdong Provincial Higher Education Institutions, College of Life Sciences, South China Agricultural University, Guangzhou 510642, China; 4 Delaware Valley University, PA 18901, USA; 5 Genetics Institute, Plant Molecular and Cellular Biology Program, University of Florida, Gainesville, FL 32610, USA

## Abstract

The molecular mechanisms of symbiosis in cultivated peanut with a ‘crack entry’ infection process are largely understudied. In this study, we investigated the root transcriptional profiles of two pairs of non-nodulating (nod−) and nodulating (nod+) sister inbred peanut lines, E4/E5 and E7/E6, and their nod+ parents, F487A and PI262090 during rhizobial infection and nodule initiation by using RNA-seq technology. A total of 143, 101, 123, 215, 182, and 289 differentially expressed genes (DEGs) were identified in nod− E4, E7 and nod+ E5, E6, F487A, and PI262090 after inoculation with *Bradyrhizobium* sp. Different deficiencies at upstream of symbiotic signaling pathway were revealed in the two nod− genotypes. DEGs specific in nod+ genotypes included orthologs to some known symbiotic signaling pathway genes, such as *NFR5, NSP2, NIN, ERN1*, and many other novel and/or functionally unknown genes. Gene ontology (GO) enrichment analysis of nod+ specific DEGs revealed 54 significantly enriched GO terms, including oxidation-reduction process, metabolic process, and catalytic activity. Genes related with plant defense systems, hormone biosynthesis and response were particularly enriched. To our knowledge, this is the first report revealing symbiosis-related genes in a genome-wide manner in peanut representative of the ‘crack entry’ species.

Cultivated peanut (*Arachis hypogaea* L.) is a crop of global importance, grown mainly in tropical and subtropical areas. It is an allotetraploid species (2n = 4x = 40, AABB; ~2.7 Gb) in genus *Arachis*, probably derived from the hybridization of two wild diploids, *Arachis duranensis* (A genome) and *Arachis ipaensis* (B genome), followed by chromosome doubling^1^,^2^,^3^. The sequences of A and B genomes are highly identical^3^. As a member of the legume family, peanut is capable of biological nitrogen fixation (BNF) through a symbiotic association with nitrogen (N) fixing bacteria, rhizobia. This symbiotic interaction results in the formation of root nodules to provide a low oxygen environment^4^. BNF is important for growth and yield of legumes and is an efficient source of N for sustainable agriculture production. In grain legumes, plants can assimilate as much as 80% of the protoplasmic nitrogen from the air^5^. Whereas in peanut, BNF only contributes about 55% of the total N the plant needs^6^, indicating that the BNF efficiency in different legumes is species-specific.

Rhizobial infection in a majority of legume species, such as the model species *Lotus japonicus, Glycine max*, and *Medicago truncatula*, follows a ‘root hair entry’ pathway, which involves intracellular infection threads (ITs) that form to guide the infection path^4^,^7^,^8^. However, for a few species such as those in the genus *Arachis* (including *A. hypogaea*) and *Stylosanthes*, rhizobial infection follows a ‘crack entry’ mechanism without the formation of ITs^9^. The ‘crack entry’ is considered as a more ancient infection mode in evolution and thus is easier to be introduced into non-legume crops than ‘root hair entry’ for improving their nitrogen fixation efficiency^10^,^11^. However, the dearth of molecular and cellular information on ‘crack entry’ limits the utilization and transfer of BNF to non-legumes.

The molecular mechanisms of symbiosis association in model legumes with ‘root hair entry’ have been extensively studied and many symbiotic genes in *L. japonicus* have been identified^12^. The symbiotic signaling pathway activates two parallel developmental processes of nodulation, bacterial infection and nodule organogenesis^7^. Before entering into the roots, rhizobia produce nod factors (NFs), a signaling molecule, which can be recognized by the host plant^7^. NF determines the specificity between the host plants and rhizobia, and initiates the infection process and the formation of nodules^13^. The NF receptor genes such as *NFR1* and *NFR5* in *L. japonicus*^14^,^15^, and *LYK3* and *NFP* from *M. truncatula*^16^,^17^, control the recognition of NFs. In addition, the receptor-like kinases SYMRK in *L. japonicus* and DMI2 in *M. truncatula* function as co-receptors for NF signaling^8^. Activated by the NF recognition process, a secondary messenger causes calcium oscillations in the nucleus responsible by nuclear membranes proteins such as POLLUX, CASTOR, and nuclear pore (NUP85, NUP133, and NENA)^8^. The calcium oscillations are decoded by the CCaMK and CYCLOPS complex at the nucleus^8^. Via transcription factors encoded by nodulation signal pathway genes (*NSP1* and *NSP2*), which further stimulate transcription factors such as nodule inception proteins (NIN) and ERN, the signals are transmitted through the symbiotic signaling pathway for nodule morphogenesis to occur^7^,^8^.

The transcriptome is a valuable resource for molecular marker development and candidate gene identification for traits of interest^18^. Many symbiosis-related genes in model legume species have been identified from high throughput transcriptome studies. A transcriptome profiling of *G. max* revealed 1,973 differentially expressed genes (DEGs) during root hair infection, including orthologs of *NFR5* and *NIN*^19^. Through microarray experiments, several metabolic genes for a phenylpropanoid pathway involved in symbiotic nitrogen fixation were identified in *L. japonicus* nodules^20^. Transcriptome profiling of *M. truncatula* root hairs discovered that rhizobial infection was associated with the expression of genes involved in cell cycle, hormone signaling, and flavonoid biosynthesis, and that the initiation of ITs requires auxin signaling regulation^21^. These studies provided molecular insights into symbiosis in legumes that use the ‘root hair entry’ mode. However, transcriptome profiling for species that utilize ‘crack entry’ has rarely been reported to date and the symbiotic pathway of ‘crack entry’ is largely unknown.

Non-nodulating (nod−) peanuts were first reported in 1979^22^. The nod− peanut lines were noticed in a F_3_ population derived from the cross between nodulating (nod+) 487A-4–1–2 (a Virginia botanical type from the University of Florida peanut breeding program) and nod+ PI262090 (a Virginia botanical type). Following this discovery, several gene inheritance models on peanut nodulation were proposed including a two-gene model^23^ and a three-gene model^24^,^25^. However, the molecular mechanisms of nodulation in peanut, a plant system with ‘crack entry’, are unaddressed. In this study, we investigated the transcriptional profiles of two pairs of “near isogenic” nod+ and nod− sister inbred lines and their nod+ parental lines during rhizobial infection and nodule initiation. The objective of this study was to discover genes related to peanut symbiosis representative of ‘crack entry’. To our knowledge, this is the first report revealing symbiosis-related genes in a genome-wide scale in cultivated peanut.

## Results

### Phenotypic response of cultivated peanut genotypes to rhizobia

**Figure 1:**
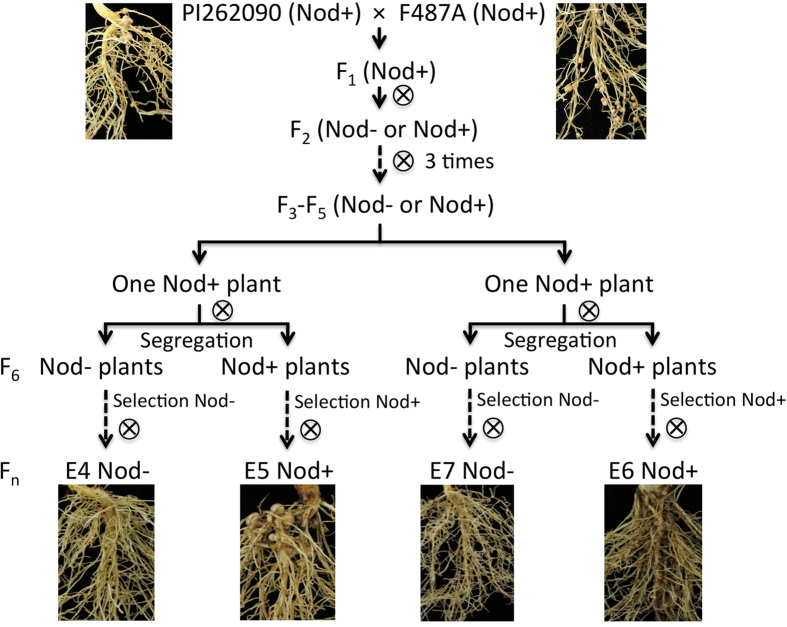
Pedigree of the six lines including PI262090, F487A, E4, E5, E6, and E7.

Six cultivated peanut lines (hereinafter also referred to as ‘genotypes’) were used in this study, including PI262090, F487A, E4, E5, E6, and E7 (Fig. 1). E4, E5, E6, and E7 are sister inbred lines originating from a cross between PI262090 (a Virginia botanical type) and F487A (a Virginia botanical type from the University of Florida peanut breeding program). E4 (nod−) and E5 (nod+) were a pair of sister inbred lines selected at the F_7_ generation from a single segregating F_6_ line. E7 (nod−) and E6 (nod+) were the other pair of sister inbred lines selected at the F_7_ generation from a different single segregating F_6_ line. For nod+ lines E5, E6, F487A, and PI262090, nodules were observed on roots inoculated with the rhizobia or ‘treatment’ plants, and absent on roots un-inoculated with the rhizobia or ‘control’ plants. For the two nod− genotypes E4 and E7, no nodules were observed for both ‘treatment’ and ‘control’ plants.

### Genome-guided assembly and annotation of novel transcripts

RNA-seq of 36 samples of the six genotypes with three replicates under ‘treatment’ or ‘control’ generated a total of 403,245,464 pairs of 150-bp reads (121.0 Gb) with an average of 11.2 million read pairs per library. By comparing the accumulated size of the sequences generated in this study to the total size of overall transcript sequences of *A. hypogaea* (109.0 Mb for both A and B genomes), the sequence generated in this study was more than 1000 times coverage of the peanut transcript sequences with an average coverage of 30.8 times per library. After trimming, 88.3% of the raw reads, including 327,078,623 read pairs and 57,906,916 un-paired ‘orphan’ reads, survived (Supplementary Table 1). The trimmed reads were mapped to the two wild diploid genomes^3^ and the overall accepted mapping rate per library ranged from 59.5% to 65.4% (Supplementary Table 1), with an average mapping rate of 62.7%. Through a genome-guided assembly, 32,571 and 297,666 transcript sequences from A and B genome (Table 1) were obtained, respectively. After removing redundancy 137,937 A genome transcript sequences remained with an N50 of 1,281 bp, and 180,509 B genome sequences remained with an N50 of 1,493 bp (Table 1). These numbers were significantly higher than the number of genes annotated in the A (36,734) and B (41,840) genomes^3^. For the A genome, a total of 70,401 (51.0%) assembled sequences matched to transcripts annotated from the reference A genome, while 87,889 (48.7%) assembled sequences matched to transcripts annotated from the B genome. Thus a total of 67,536 (49.0%) and 92,620 (51.3%) additional or novel transcript sequences in A and B genomes respectively were identified in this study. Additionally, 3,892 and 5,864 sequences from the A and B genomes respectively were full-length transcripts.

To explore the potential functions of these novel transcript sequences, they were blasted against the ‘nr’ database at the NCBI. In total 11,624 (17.2%) and 18,888 (20.4%) sequences from A and B genomes respectively had hits to the ‘nr’ database. A total of 1,005 and 1,016 gene ontology (GO) terms were assigned to 3,922 and 5,843 sequences from A and B genomes, respectively. After searching the PlnTFDB database, for the A genome, a total of 3,355 sequences were transcription factor-encoding transcripts corresponding to 40 transcription factor families and 11 other transcription factor regulators. For the B genome, 5,460 were transcription factor-encoding sequences belonging to 39 transcription factor families and 12 other transcription factor regulators. Using ‘Preloaded small RNAs’ at psRNATarget website, a total of 5,878 miRNA accessions were identified to target 11,668 (17.3%) novel transcript sequences from the A genome, and 6,197 miRNA accessions targeting 15,470 (16.7%) novel sequences from the B genome. In addition, there were 173 KEGG Orthology (KO) terms assigned to 266 sequences in the A genome and 191 KO terms assigned to 291 sequences in the B genome.

### Identification of differentially expressed genes

The DEGs were determined between ‘control’ and ‘treatment’ samples of each genotype. For the nod− genotypes (E4 and E7), most of the DEGs should be unrelated to nodule organogenesis. In total, 233 genes (Supplementary Table 2) were differentially expressed in at least one nod− genotype, including 143 genes in E4, 101 genes in E7 (Table 2), and 11 genes (6 up-regulated and 5 down-regulated) identified in both nod− genotypes. For the nod+ genotypes, a total of 123, 215, 182, and 289 genes in E5, E6, F487A, and PI262090, respectively, were differentially expressed between ‘control’ and ‘treatment’ samples. There were 21 genes showing either up-regulation or down-regulation in both nod+ and nod− genotypes (Supplementary Table 2). After subtracting the DEGs in nod− genotypes (E4 and E7) from the DEGs in nod+ genotypes, in total 307 genes were up-regulated specifically in nod+ genotypes (E5, E6, PI262090, F487A) in response to the infection of bradyrhizobia, while 244 genes were down-regulated.

**Figure 2:**
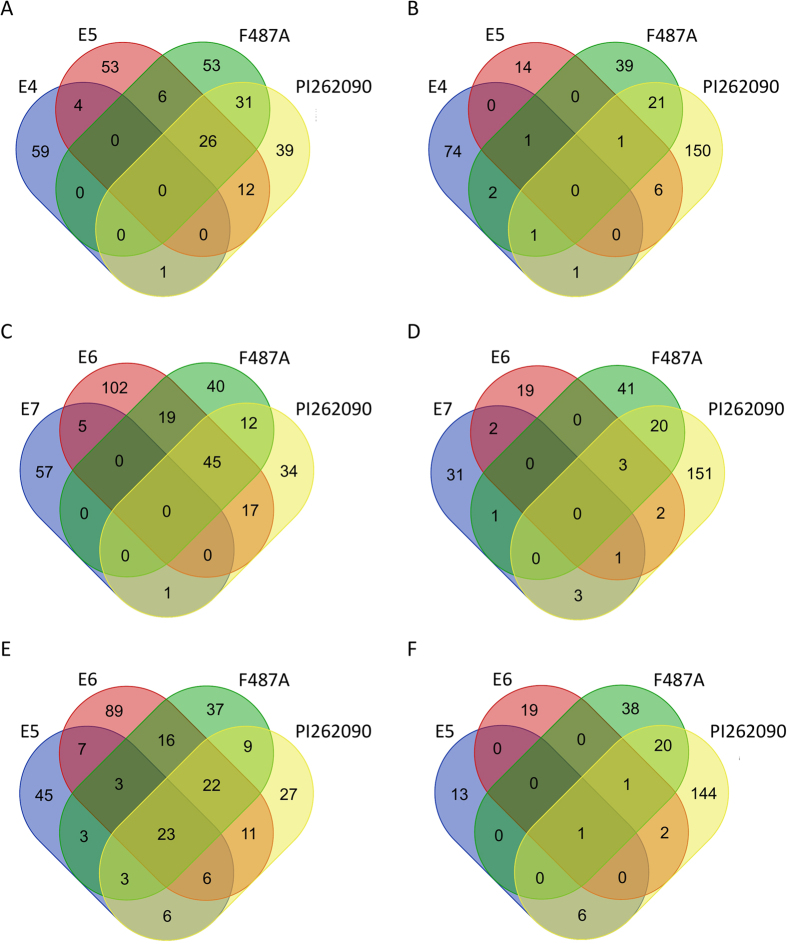
Comparison of differentially expressed genes identified in the six genotypes, PI262090, F487A, E4, E5, E6, and E7. (**A,C,E**) Up-regulated genes. (**B,D,F**) Down-regulated genes. (**A–B**) Comparison of genes between the non-nodulating E4 and three nodulating genotypes. (**C–D**) Comparison of genes between the non-nodulating E7 and three nodulating genotypes. (**E–F**) Comparison of genes specifically differentially expressed in four nodulating genotypes.

**Figure 3:**
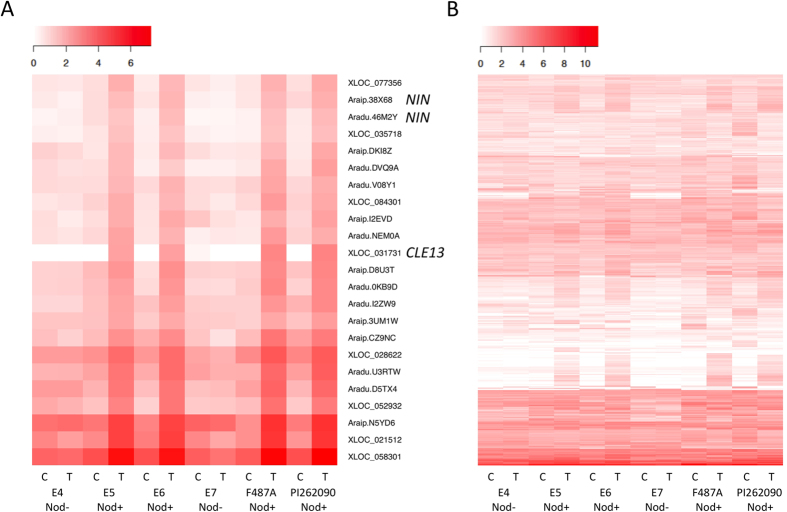
Heat maps of the differentially expressed genes specifically in nodulating genotypes of the six genotypes inoculated and un-inoculated with rhizobia. Transformed FPKM values were used. “White’ color indicates no expression or low expression level; and “red” color indicates high expression level. ‘C’ indicates ‘control’; ‘T’ indicates ‘treatment’. (**A**) Expression profiles of the 23 core up-regulated genes shared by all four nod+ genotypes. (**B**) Expression profiles of all 544 differentially expressed genes.

The DEGs from the six genotypes were compared with each other to investigate which genes failed to respond to the infection of rhizobia in nod− genotypes as well as those normally involved in nodulation in nod+ genotypes (Fig. 2). Most of the DEGs from nod+ genotypes were not shared with either of the two nod− genotypes. E4 only shared 10 DEGs (5 up-regulated and 5 down-regulated) with its nod+ controls (Fig. 2A,B). E7 shared 13 DEGs (6 up-regulated and 7 down-regulated) with its nod+ controls (Fig. 2C,D). The DEGs from the four nod+ genotypes were also compared with each other. Among the up-regulated genes, 23 DEGs were shared by all of the nod+ genotypes (Fig. 2E), showing an obvious up-regulation pattern in nod+ genotypes (Fig. 3A). On the contrary, only one gene was down-regulated in all four nod+ genotypes (Fig. 2F). The genes that were differentially expressed in at least one nod+ genotype were subjected to subsequent functional analysis. In summary, 544 unique genes with different expression profiles (Fig. 3B) were identified, including 511 genes that showed no regulation in nod− genotypes (Supplementary Table 2), but were either up-regulated (293 genes) or down-regulated (225 genes) in nod+ genotypes. Among these DEGs, seven genes showed up-regulation in one nod+ genotype and down-regulation in another nod+ genotype, which may be caused by different genetic backgrounds of nod+ genotypes. The remaining 33 genes of the 544 genes were regulated in nod− genotypes, but were oppositely regulated in at least one nod+ genotype.

### Gene Ontology enrichment and functional classification of differentially expressed genes

**Figure 4:**
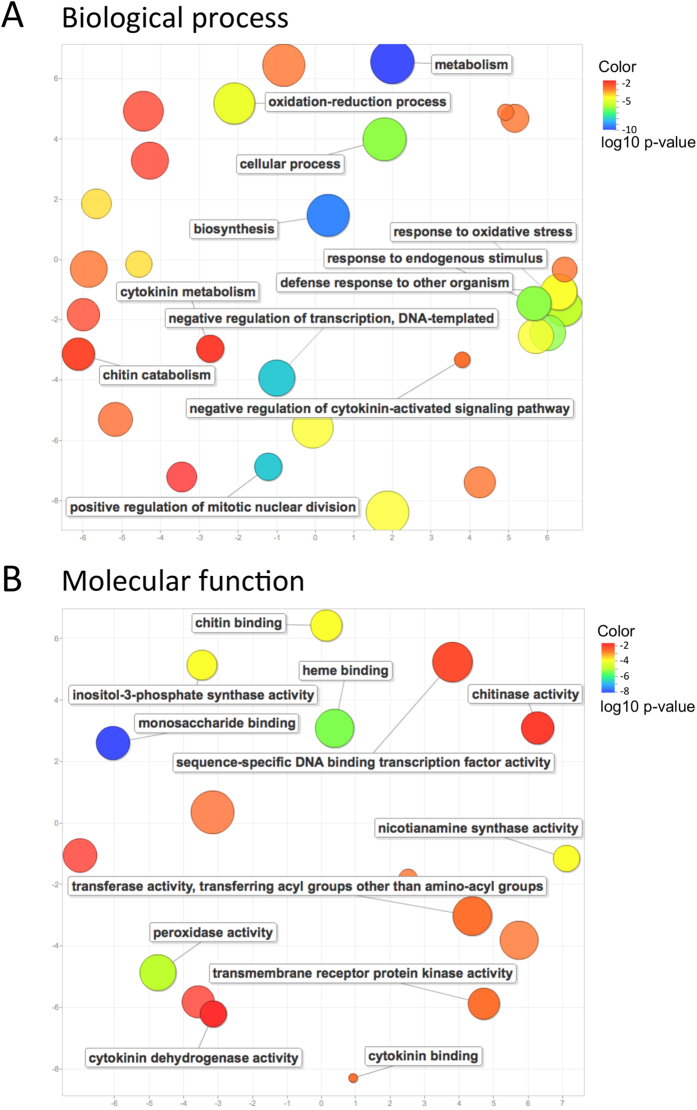
Significantly enriched gene functions in differentially expressed genes specifically in nodulating genotypes. Enriched GO terms were projected onto a 2D semantic space. (**A**) Enriched GO categories in biological process. (**B**) Enriched GO categories in molecular function.

GO enrichment analysis was performed to identify processes and functions over-represented in DEGs. Four GO terms were enriched in DEGs from nod− genotypes, including oxidation-reduction process, oxidoreductase activity, flavin mononucleotide (FMN) binding, and plastid. When looking at DEGs specifically identified in nod+ genotypes, a total of 54 GO terms were significantly enriched (Supplementary Table 3). The most significantly enriched GO term was oxidation-reduction process, followed by metabolic process and catalytic activity. Particularly, for the DEGs identified in nod+ genotypes, a cluster of GO terms related with the plant defense system, such as “response to oxidative stress”, “response to endogenous stimulus”, and “defense response to other organism” were observed (Fig. 4A). The regulation of transcription, including “negative regulation of transcription, DNA-templated”, was active in nod+ genotypes upon infection of bradyrhizobia. Additionally, “cytokinin metabolism” and “negative regulation of cytokinin-activated signaling pathway” in biological process (Fig. 4A), “cytokinin binding” and “cytokinin dehydrogenase activity” in molecular function (Fig. 4B) were observed, indicating the role of cytokinin in the regulation of nodulation. Furthermore, many chitin related GO terms were observed, including “chitin catabolism” in biological process, “chitin binding” and “chitinase activity” in molecular function (Fig. 4B). The GO term “positive regulation of mitotic nuclear division” was observed (Fig. 4A), potentially involved in cell division during nodule formation.

Based on all of the annotations, the DEGs in nod+ genotypes were classified into genes involved in cell cycle, phenylpropanoids, plant hormones, transcription factor genes, and diseases resistance genes (Supplementary Table 2). Specifically, 13 DEGs were orthologous to known cyclin-dependent kinase (CDK) genes, which regulate the progression of the cell cycle. For most of the 34 DEGs involved in phenylpropanoid biosynthesis, an up-regulation was observed in at least one nod+ genotype. A total of 27 DEGs (14 up-regulated and 13 down-regulated) were found to be involved in response and signal transduction of plant hormones, including auxin, cytokinin, ethylene, gibberellic acid (GA), jasmonic acid (JA), and strigolactone (SL). Surprisingly, 14 (51.9%) of these genes were ethylene related genes, encoding ethylene-responsive transcription factors. Six AP2-like ethylene-responsive transcription factor genes showed up-regulation in at least one nod+ genotype, while the remaining ethylene-responsive transcription factor genes showed only down-regulation in at least one nod+ genotype. Both up-regulation and down-regulation were observed for the auxin and GA related genes. Out of the three cytokinin oxidase/dehydrogenase genes, two were up-regulated in all four nod+ genotypes upon infection, while the other was only up-regulated in F487A. Only down-regulation was observed for the two JA related genes. In addition, 14 other transcription factor genes (6 up-regulated and 8 down-regulated) and six disease resistance genes (up-regulated) were among the DEGs.

### Identification of peanut orthologs in model legumes

To investigate the common genes involved in nodulation between *A. hypogaea* with ‘crack entry’ infection mode and the two model legumes, *L. japonicus* and *M. truncatula* with ‘root hair entry’ mode, orthologous groups were identified among genes from these three species using OrthoMCL. The 440 out of 544 DEGs specifically in nod+ genotypes were assigned to 315 gene families (Supplementary Figure 1A), out of which 277 (87.9%) were shared by the two model species. Eight gene families (containing 19 genes) and 103 unassigned genes were specific to *A. hypogaea* (Supplementary Table 2). Among these 122 peanut-specific genes, 22 were annotated with functions, while most of other genes had unknown functions. When considering all the *A. hypogaea* genes, a total of 12,123 gene families accounting for only 38.2% of *A. hypogaea* gene families were shared with the two model species (Supplementary Figure 1B), much lower than the shared portion of DEGs. *L. japonicus* and *M. truncatula* shared 13,333 gene families, accounting for 80.4% and 84.9% of their total gene families, respectively (Supplementary Figure 1A).

### Identification and expression analysis of orthologous nodulation-related genes

A total of 125 peanut genes were identified as orthologs of previously published nodulation-related genes by using both OrthoMCL and reciprocal BLASTp, including most of the key symbiotic signaling pathway genes *NFR1, NFR5, SYMRK, NIN, ERN1, NSP2, CCaMK, CYCLOPS, CASTOR, NUP85, NUP133, LHK1, RAM1, RAM2*, and *HAP2.1* (Supplementary Table 4). Out of these 125 peanut orthologs, 12 were DEGs specifically in nod+ genotypes, corresponding to six types of nodulation-related genes, whose expression were further investigated (Table 3). Specifically, the peanut ortholog of *NFR5*, essential for NF perception^14^, Araip.NL2P7, was found to be up-regulated in nod+ E6 and PI262090 upon infection of bradyrhizobia, while it showed a significantly low level of expression in the two nod− genotypes. The expression of this *NFR5* ortholog was validated by qRT-PCR (Supplementary Table 5). The peanut ortholog of *nodulation-signaling pathway 2 (NSP2*), XLOC_071109, was up-regulated in nod+ E6 upon infection, while it showed very low level of expression in the two nod− lines. Orthologs of two early nodulin genes that are induced by NFs were differentially expressed, including *RIP1*, encoding a peroxidase, and *ENOD16*, belonging to the family of phytocyanin-related early nodulins^26^,^27^. The two peanut orthologs of *RIP1*, Aradu.X9PNA and Araip.SHV7N, were down-regulated in nod+ genotypes upon infection, but it showed no significant change in expression in nod− genotypes. The two orthologs of *ENOD16*, Aradu.D9DNB and Araip.AZY85, were up-regulated in nod+ genotypes upon infection, while it showed a low level of expression for all the other situations. The genes Aradu.46M2Y and Araip.38X68, orthologous to the *NIN* gene, related with nodule organogenesis^28^, were up-regulated in all nod+ genotypes upon infection. The orthologs of *ERN1*, a transcriptional regulator of early nodulin *ENOD11*^29^, showed up-regulation in nod+ F487A upon infection. In addition, the two orthologs of *CLE13*, XLOC_075006 and XLOC_031731, involved in controlling the number of nodules and systemic signals^30^, were up-regulated in at least three nod+ genotypes upon infection, while it showed a low level of expression in all other situations.

### qRT-PCR validation

To validate the gene expression levels of the DEGs, 10 DEGs between E6 and E7 were selected for validation using qRT-PCR. Among these genes, eight were confirmed to be up-regulated with more than a two-fold change in E6 (Supplementary Table 5). For the remaining two genes, gene ‘Aradu.T3S5X’ was confirmed to be down-regulated in E6, but with less than a two-fold change; gene ‘Aradu.62DXS’ was confirmed to be up-regulated in E6, again with less than a two-fold change (Supplementary Table 5). The results of qRT-PCR agreed well with most findings from RNA-seq analysis.

## Discussion

The available peanut ancestors’ genomes have accelerated research on transcriptional studies by providing valuable genome references. However, the genome annotation could be ameliorated by discovery of novel transcripts. In this study, a total of 67,536 and 92,620 novel transcript sequences aligned to the reference genomes near perfectly were identified from A and B genomes, respectively, which were most likely the transcripts un-annotated in the reference genomes. Out of these novel transcript sequences, 3,892 and 5,864 in A and B genomes, respectively were full-length transcripts, which would contribute to the number of gene models annotated from the reference genomes. Specifically, 159 DEGs related to nodulation were discovered among these novel transcripts. However, some of the transcripts could be cultivated peanut-specific, which were pseudogenes or non-transcripts in the ancestors’ genomes. The A and B reference genomes were highly identical with a median DNA identity of 93.11%^3^. Thus most of the transcript sequences from the A genome would have a copy in the B genome. In this study, the homoeologous gene pairs with an identity of less than 98.7% should be distinguished during alignment since the default maximum mismatches was ‘2’ in 150 bp read alignments. Moreover, we found that more than 99% of homoeologous gene pairs in DEGs had an identity of less than 98.7%. Therefore the concern regarding accuracy of expression level estimation for genome-specific genes was diminished in this study.

The molecular mechanisms of nodulation in ‘crack entry’ species have rarely been reported. Our research filled this knowledge gap by investigating the transcriptome profiles of cultivated peanut roots after bradyrhizobia infection. In this study, we analyzed transcriptome of whole peanut root at 5 DAI. Since the first nodule was typically observed at 10 DAI in our system and gradually more nodules emerged at later dates, we believe that the transcripts captured at 5 DAI of the whole root should be involved in the processes prior to and during nodule organ initiation as multiple events including rhizobia recognition, infection, and nodule organogenesis were presumably happening at different positions of the root at 5 DAI. In this study, we only focused on the strictly identified DEGs (with high stringency of 2 fold changes and FDR < 0.05) responding to inoculation at 5 DAI to obtain a quick glimpse of the molecular responses during early stages of peanut nodulation. This, to our knowledge, is the first attempt to investigate the molecular response during nodulation in a plant system with ‘crack entry’, an ancient process of bacteria and host interaction.

We are not certain whether the nod− genotypes in this study were dysfunctional in the process of rhizobia recognition, rhizobia infection, or nodule organogenesis. By looking at the number of DEGs identified in each genotype, we noticed that the DEG numbers in nod− E4 and E7 were not dramatically less than their nod+ sister lines specifically in nod− E4 (Table 2) indicating that both nod− genotypes actively responded to the rhizobia infection. This deduction can be further confirmed by the observation that 21 DEGs in nod− genotypes were also DEGs in nod+ genotypes (Supplementary Table 2). The nod− genotypes in this study provided an important reference to filter out the DEGs simply just responding to rhizobia infection. Thus we were able to select the DEGs involved in nodule organogenesis and some DEGs involved in the symbiotic signaling pathway if the nod− genotypes had genetic defects at an early stage of rhizobia infection process.

However, when comparing DEGs between nod− genotypes and nod+ genotypes (Fig. 2A–D), it was apparent that most of the genes normally involved in nodulation had no response to rhizobia infection in nod− E4 and E7. Among these DEGs were several important symbiotic signaling pathway genes, including orthologs of *NFR5, NSP2, NIN*, and *ERN1*. Specifically, the two peanut orthologs of *NIN* were up-regulated in all four nod+ genotypes, but were not regulated in either E4 or E7 (Table 3). In *M. truncatula*, the *NIN* mutants were blocked in infection and not able to form nodules^28^. This strongly indicated that the symbiosis signaling pathway was activated in nod+ genotypes but not in nod− E4 or E7. The two nod− mutants are blocked either at the symbiotic signaling stage in *NIN* or upstream of *NIN*, or even earlier process of nodulation. Based on our analysis, E4 and E7 probably have different genetic defects due to several reasons. Firstly, only 11 (4.7%) DEGs were shared between E4 and E7 out of the 233 DEGs identified in these two nod− genotypes, indicating that E4 and E7 had a different response to rhizobia infection. Secondly, we have developed two F_2_ mapping populations derived from two crosses (E4 & E5; E6 & E7). The segregation ratios of nod+ to nod− are different (unpublished data), which is a clue that the inheritance of nodulation in these two populations is different or the genetic defects of E4 and E7 are different. A further investigation based on results from this study is needed to uncover the genetic mechanisms of non-nodulation.

According to OrthoMCL analysis, 87.9% of the peanut gene families of DEGs were shared with *L. japonicus* and *M. truncatula*, while only 38.2% of the whole genome-wide gene families were shared among the three legume species (Supplementary Figure 1A). The conservativeness of DEGs implied certain common genetic mechanisms of nodule organogenesis in legume species. The transcriptome study of *M. truncatula* has reported 370 conserved symbiosis genes in response to rhizobia, which were shared with *G. max*^21^, indicating they were recruited before the divergence of *M. truncatula* and *G. max*. As a legume species, *A. hypogaea* also shared many symbiosis genes with other model legumes, as demonstrated by the discovery that most of the genes in the common symbiotic signaling pathway published previously had their orthologs in peanut (Supplementary Table 4).

In a common symbiotic signaling pathway characterized in model legume species, the first step is the recognition of NFs by LysM receptor-like kinases^8^. There are two forms of such kinases, *NFR1* (or *LYK3* in *M. truncatula*) and *NFR5*. The LysM domain of *NFR5* at least partially determines the specificity of NF recognition^31^. With a non-functional kinase domain, *NFR5* has been reported to couple with *NFR1* as heterodimers in NF recognition signal transduction^32^. Two *NFR5* orthologs were identified in peanut genomes with complete LysM receptor-kinase domains and only one of them, Araip.NL2P7, was identified as a DEG, which was up-regulated in nod+ genotype in response to rhizobial indicating its expression induction during the nodulation process. Two peanut *NFR1* orthologs were identified in the peanut genomes, but none of them had complete LysM receptor-kinase domains and none of them were DEGs in any genotypes. These results indicate that the peanut *NFR5* ortholog may play a similar role in recognizing NFs and might lead to downstream signaling activation^8^. In this study, the downstream symbiosis signaling pathway genes, *NSP2, NIN,* and *ERN1* were found differentially expressed in nod+ lines, which indicated their role in peanut nodulation.

In addition to the symbiotic pathway genes, plant hormone related genes also play an essential role in regulating rhizobial infection and nodule organogenesis. Ethylene has been found to inhibit the NF signal transduction pathway in *M. truncatula*, and may play as a secondary signal in response to N status in plants to regulate nodulation^33^. In *M. truncatula*, an ethylene response factor (ERF) gene belonging to the AP2/EREBP family was up-regulated during nodulation^34^. Similarly, the *LjERF1* gene, also belonging to the AP2/EREBP family, had a positive regulatory role in nodulation in *L. japonicus*^35^. In our study, six AP2-type ERF genes showed up-regulation in nod+ genotypes. One of them (Aradu.0KB9D) was up-regulated in all four nod+ genotypes and probably played a similar role in nodulation with the aforementioned two ERF genes in *M. truncatula* and *L. japonicus*. In addition, two JA-responsive genes were down-regulated in a nod+ genotype after infection. JA is a hormone involved in plant defense system. The production of JA is typically repressed during normal symbiosis process to allow the rhizobia to infect the host. The JA-related responses were found to be repressed by NFs^21^. Therefore, this JA repression mechanism most likely exists in peanut nodulation. In white clover, auxin has been reported to play an important role during the process of nodule organogenesis through a transient inhibition of auxin transport. Auxin responsive genes were initially down-regulated and then up-regulated at the inoculation site^36^. In our study, the gene orthologous to *Gretchen Hagen3.1 (GH3.1)*, which encodes a putative indole-3-acetic acid-amido synthetase, showed up-regulation in nod+ genotypes after infection, implying at least an increased activity of auxin in peanut roots during nodulation. During early symbiosis signaling, the production of GA tends to increase due to the NF signaling^21^. This still held true in peanut, as three peanut genes related with GA biosynthesis were all up-regulated in nod+ genotypes after infection. A recent study in *L. japonicus* has revealed the role of *cytokinin oxidase/dehydrogenase3 (Ckx3*), which is induced by NF during the initiation of nodules^37^. In addition to the above plant hormone genes, SL also played a role during the rhizobial infection process, as a gene orthologous to *carotenoid cleavage dioxygenase 8* related with strigolactone biosynthesis, was up-regulated in a nod+ genotype after infection. This result was consistent with previous findings in *M. truncatula*^21^.

The *CLE* genes were known to encode secreted peptides whose signaling was integrated with phytohormone signaling to regulate numerous biological processes in response to environmental stimuli^38^, in addition to nodulation in legumes^39^. These peptides were perceived by LRR-RLK genes, establishing a CLE-RLK module to convey the signaling cascades intracellularly and extracellularly^40^, which was further demonstrated in our study. The two *CLE13* orthologs in peanut were up-regulated in nod+ genotypes upon infection, most likely induced by plant hormones (ethylene and cytokinin) and interacted with the two LRR-RLK genes that were DEGs in nod+ genotypes.

In summary, as a member of the legume family, *A. hypogaea* with a “crack entry” infection mode shared a considerable number of nodulation related genes with model legumes with ‘root hair entry’. To reveal the mechanisms specific to the ‘crack entry’ mode of infection, additional studies are needed to focus on the 122 peanut-specific DEGs, including the eight gene families and the 102 unassigned genes that shared no homology with genes from the two model legumes, as well as the 21 DEGs showing similar differential expression in both nod− and nod+ genotypes. This study is a pioneer study that has revealed nodulation related genes for a ‘crack entry’ species in a genome-wide manner.

## Methods

### Plant materials and inoculation

There were six cultivated peanut genotypes used in this study, including PI262090, F487A, E4, E5, E6, and E7 (Fig. 1). Approximately 100 seeds of each genotype were sterilized in 0.1% HgCl_2_ solution for seven min, and then washed three times using sterilized ddH_2_O for five min each time. The sterilized seeds were soaked in sterilized ddH_2_O for two days, and then were transferred into a sterilized germination box. After four days, the germinated seeds were transferred to Ziploc bags with germination paper inserts and 40 ml 25% Hoagland’s solution without N. When the root grew to 6–7 cm, a one ml suspension of a single strain of *Bradyrhizobium spp.*, Lb8 (A_600_  =  0.05–0.1) isolated in our laboratory from peanut nodules was applied to the root in the Ziploc bag for inoculation. Three plants of each genotype (treatment) were inoculated with the rhizobia and three plants of each genotype un-inoculated were used as treatment ‘controls’. At 5 DAI, when the nodule primordia were presumably emerging and rhizobia infection was occurring (nodules were first visible to the naked eye at 10 DAI), the primary root of the top 10 cm from each plant was cut from three treated and three untreated plants and immediately put into liquid nitrogen for RNA extraction. In the meantime, additional plants of each genotype that were inoculated and un-inoculated were not sampled and kept growing for nodule formation confirmation.

### RNA extraction and library construction

Total RNA was extracted using TRizol (Invitrogen) and purified using a Qiagen RNeasy kit. The cDNA library construction was performed using the NEBNext Ultra RNA Library Prep kit. In total, 36 libraries (6 genotypes × 2 treatment levels × 3 replicates) were constructed and subsequently sequenced using Illumina NextSeq 500 at the Interdisciplinary Center for Biotechnology Research (ICBR) at the University of Florida.

### Sequence reads trimming and alignment

The raw reads from RNA-seq were trimmed by using Trimmomatic^41^ to remove the adapter and low quality sequences. The read quality was assessed using FastQC^42^. The RNA-seq data analysis pipeline followed the protocol described by Cole Trapnell^43^. Tophat2^44^ was used for alignment allowing one mismatch in the 20 bp seed and other parameters setting to default. The A and B genomes^3^ from the two wild peanut diploid ancestors were used as the reference for alignment. GFF files for both genomes were used to guide alignment.

### Genome-guided assembly and functional annotation

The alignment files of the 36 samples resulting from Tophat2 were combined and input into Trinity (–max_memory 64G –genome_guided_max_intron 10000 –CPU 16)^45^ for a genome-guided assembly. To remove redundancy, the assembled transcript sequences from each genome were clustered using CD-HIT-EST (-c 0.95 -n 9 -T 0 -M 0 -r 1)^46^. The resulting reference –based assembly was compared to its respective transcript files from annotated reference genomes by using Blat (-t = dna -q = dna -out = blast9)^47^. An e-value cutoff of ‘1e-05’ was used to determine a hit. The transcript sequences without hits were treated as novel transcripts compared to the genome annotation and were subsequently annotated in this study.

Novel transcript sequences were compared to the ‘nr’ database at NCBI by BLASTX (-b 20 -v 20 -p blastx -e 1e-5 -m 7). GO terms were assigned using Blast2Go^48^. Full-length transcripts were predicted using TargetIdentifier (http://bioinformatics.ysu.edu/tools/TargetIdentifier.html). The Plant Transcription Factor Database (PlnTFDB) (http://plntfdb.bio.uni-potsdam.de/v3.0/) was used to search for potential transcripts involved in transcriptional control (e-value 1e-6 for BLAST). To identify important pathways involved by the novel transcripts, the transcripts were assigned to the Kyoto Encyclopedia of Genes and Genomes (KEGG) pathways using the web server (http://www.genome.jp/kaas-bin/kaas_main) against the *Arabidopsis thaliana* and *Glycine max* gene datasets using the bi-directional best hit (BBH) method. The transcript sequences were translated into protein sequences using OrfPredictor^49^.

### Identification of differentially expressed genes, GO enrichment analysis, and ortholog search

The DEGs were identified using Cufflinks and Cuffdiff^43^ with cutoff: fold change > =  2, FDR< 0.05. The DEGs were identified through the comparison of gene expression between ‘control’ and ‘treatment’ samples for each genotype. GO terms for gene models available in genome annotation were directly retrieved from the ‘GFF’ file downloaded at PeanutBase website (http://peanutbase.org). GO enrichment analysis for DEGs was performed using FatiGO software^50^ available at Babelomics5 (http://babelomics.org). The enriched GO terms were visualized using REVIGO^51^. The peanut protein sequences were compared with the protein sequences of *M. truncatula* (Mt4.0v1)^52^ and *L. japonicus* (TGI database, http://compbio.dfci.harvard.edu/tgi/) that are two widely used model legume systems. OrthoMCL software^53^ was used to detect orthologous gene groups (e-value 1e-5 for all-versus-all BLASTp, inflation value 1.5). To identify peanut orthologous genes, a comprehensive literature search^7^,^12^,^13^,^19^,^21^,^54^ for symbiosis related genes was carried out. The protein sequences of these genes were retrieved either from the original publication or from NCBI (http://www.ncbi.nlm.nih.gov). The collected protein sequences were compared to the protein sequences of peanut genes using a combination of OrthoMCL and reciprocal BLASTp following the reciprocal best hits method as previously described^55^.

### qRT-PCR validation

Primers were designed using BatchPrimer3 v1.0 (http://probes.pw.usda.gov/batchprimer3/). The primer sequences were aligned to A and B genomes using Bowtie^56^ (-v 2 -I 100 -X 1000) to make sure they had a single hit in the peanut genomes. RNA samples of E6 and E7 from roots at 5 DAI were extracted using TRIzol (Amnbion, USA) following the manufacturer’s instructions. cDNA was synthesized from 1 μg of DNaseI (NEB, England) treated RNAs with the SuperScript^®^ III First-Strand Synthesis System for RT-PCR kit (Invitrogen, Carlsbad, USA). Quantitative PCR was performed with two biological replicates and each containing three technical replicates for each sample using the Power SYBR^®^ Green PCR Master Mix kit (Appliedbiosystems, USA). The real time PCR was detected with CFX96 Real-Time PCR Detection System (Bio-Rad). The cDNA levels of target genes were normalized to the endogenous reference gene *AhUbiquitin 2*^57^.

### Data availability

The raw fastq files for the 36 libraries were deposited in the Sequence Read Archives (SRA) of NCBI under accession number SRP093688, BioProject PRJNA354154, and BioSample SAMN06041692-SAMN06041727.

## Additional Information

**How to cite this article**: Peng, Z. *et al*. Transcriptome profiles reveal gene regulation of peanut (*Arachis hypogaea* L.) nodulation. *Sci. Rep.* **7**, 40066; doi: 10.1038/srep40066 (2017).

**Publisher's note:** Springer Nature remains neutral with regard to jurisdictional claims in published maps and institutional affiliations.

## Supplementary Material

Supplementary Information

Supplementary Table 2

Supplementary Table 4

## Figures and Tables

**Table 1:** Statistics of genome-guided assembly of the transcriptome sequences using Trinity.

	Raw transcripts from Trinity		CD-HIT-EST	
	A genome	B genome	A genome	B genome
No. of assembled sequences	232,571	297,666	137,937	180,509
Average length (bp)	641.7	689.2	777.2	828.5
Minimum length (bp)	201	201	201	201
Maximum length (bp)	16,879	14,792	16,879	14,792
N50 (bp)	1,016	1,215	1,281	1,493

**Table 2:** Differentially expressed genes between samples inoculated with rhizobia and samples un-inoculated.

Genotype	Nodulation phenotype	DEGs with fold change > = 2, FDR < 0.05
E4	Nod−	143
E5	Nod+	123
E6	Nod+	215
E7	Nod−	101
F487A	Nod+	182
PI292060	Nod+	289

**Table 3:** Differential expression status of the differentially expressed genes in nodulating genotypes with orthologs to previously characterized symbiosis genes.

ID	Orthologous symbiosis-related genes	Gene name	Chromosome	E4	E5	E6	E7	F487A	PI262090
XLOC_002328	ENOD16	Aradu.D9DNB	Aradu.A01	no	no	UP**	no	no	UP**
XLOC_003129	RIP1	Aradu.X9PNA	Aradu.A01	no	DOWN*	no	no	no	DOWN**
XLOC_025526	ERN1	XLOC_025526	Aradu.A06	no	no	no	no	UP**	no
XLOC_027005	NIN	Aradu.46M2Y	Aradu.A07	no	UP**	UP**	no	UP**	UP**
XLOC_031731	CLE13	XLOC_031731	Aradu.A08	no	UP**	UP**	no	UP**	UP**
XLOC_043348	ENOD16	Araip.AZY85	Araip.B01	no	no	no	no	UP**	no
XLOC_039690	RIP1	Araip.SHV7N	Araip.B01	no	DOWN**	no	no	no	DOWN**
XLOC_050005	NIN	Araip.38X68	Araip.B03	no	UP**	UP**	no	UP**	UP**
XLOC_057941	NFR5	Araip.NL2P7	Araip.B05	no	no	UP**	no	no	UP**
XLOC_066947	ERN1	XLOC_066947	Araip.B06	no	no	no	no	UP**	no
XLOC_071109	NSP2	XLOC_071109	Araip.B07	no	no	UP**	no	no	no
XLOC_075006	CLE13	XLOC_075006	Araip.B08	no	UP**	no	no	UP**	UP**

Note: The last six columns show the comparison of ‘control’ and ‘treatment’ samples for the six genotypes. ‘UP’ indicates up-regulation; ‘DOWN’ indicates down-regulation. ‘*’: less than two-fold change; ‘**’ two-fold change or more.
