# Supplementary material for: Transcriptome profiles reveal gene regulation of peanut (Arachis hypogaea L.) nodulation
Source: Sci Rep. 2017 Jan 6;7:40066. doi: 10.1038/srep40066 (PMC5216375; doi:10.1038/srep40066)
Supplement: Supplementary Information [file srep40066-s1.pdf]

Supplementary Information

Transcriptome profiles reveal gene regulation of peanut (*Arachis hypogaea* L.) nodulation

Ze Peng, Fengxia Liu, Liping Wang, Hai Zhou, Dev Paudel, Lubin Tan, James Maku, Maria Gallo, Jianping Wang*

*Corresponding author, Email: wangjp@ufl.edu

Supplementary Table 1. Summary of trimming and alignment of reads to A and B genomes in each library.

Library name	Genotype	Sample type	Input read pairs	Both surviving	Forward only surviving	Reverse only surviving	Dropped	Overall alignment rate (%)
1-FCK2	F487A	control	13058247	10501464 (80.42%)	1394880 (10.68%)	488747 (3.74%)	673156 (5.16%)	63.30
2-FCK3	F487A	control	11499086	9359144 (81.39%)	1198046 (10.42%)	420697 (3.66%)	521199 (4.53%)	64.10
3-FCK6	F487A	control	10882306	8824631 (81.09%)	1267248 (11.65%)	344660 (3.17%)	445767 (4.10%)	64.40
4-FT2	F487A	treatment	11214812	9000395 (80.25%)	1256070 (11.20%)	413764 (3.69%)	544583 (4.86%)	62.70
5-FT3	F487A	treatment	7606390	6058212 (79.65%)	889256 (11.69%)	283970 (3.73%)	374952 (4.93%)	60.20
6-FT5	F487A	treatment	10253258	8432203 (82.24%)	1099681 (10.73%)	334621 (3.26%)	386753 (3.77%)	63.20
7-PICK1	PI262090	control	9938028	8097290 (81.48%)	1083686 (10.90%)	326370 (3.28%)	430682 (4.33%)	64.70
8-PICK2	PI262090	control	12130540	9932860 (81.88%)	1189125 (9.80%)	469750 (3.87%)	538805 (4.44%)	62.00
9-PICK3	PI262090	control	11574363	9367944 (80.94%)	1300933 (11.24%)	399997 (3.46%)	505489 (4.37%)	62.70
10-PIT2	PI262090	treatment	11940182	9449775 (79.14%)	1417703 (11.87%)	437991 (3.67%)	634713 (5.32%)	61.70
11-PIT3	PI262090	treatment	10818936	8388088 (77.53%)	1442199 (13.33%)	390105 (3.61%)	598544 (5.53%)	60.10
12-PIT4	PI262090	treatment	9436075	7676177 (81.35%)	1053309 (11.16%)	320496 (3.40%)	386093 (4.09%)	63.30
13-E4CK1	E4	control	11565431	9452894 (81.73%)	1148418 (9.93%)	442738 (3.83%)	521381 (4.51%)	64.00
14-E4CK3	E4	control	10387342	8596082 (82.76%)	1053467 (10.14%)	351167 (3.38%)	386626 (3.72%)	65.30

15-E4CK4	E4	control	9182335	7426986 (80.88%)	1083233 (11.80%)	290887 (3.17%)	381229 (4.15%)	62.50
16-E4T1	E4	treatment	11853554	9574662 (80.77%)	1286423 (10.85%)	435063 (3.67%)	557406 (4.70%)	59.50
17-E4T2	E4	treatment	9784316	7779661 (79.51%)	1167380 (11.93%)	355354 (3.63%)	481921 (4.93%)	60.50
18-E4T3	E4	treatment	9829616	8093329 (82.34%)	1056395 (10.75%)	311953 (3.17%)	367939 (3.74%)	63.00
19-E5CK1	E5	control	11947025	9795657 (81.99%)	1180245 (9.88%)	449225 (3.76%)	521898 (4.37%)	64.50
20-E5CK3	E5	control	12307105	9950400 (80.85%)	1382051 (11.23%)	430433 (3.50%)	544221 (4.42%)	61.20
21-E5CK4	E5	control	11063904	9000109 (81.35%)	1243270 (11.24%)	368318 (3.33%)	452207 (4.09%)	62.20
22-E5T2	E5	treatment	16053969	13121724 (81.74%)	1555306 (9.69%)	609893 (3.80%)	767046 (4.78%)	63.80
23-E5T3	E5	treatment	11834264	9786796 (82.70%)	1150536 (9.72%)	413858 (3.50%)	483074 (4.08%)	63.40
24-E5T4	E5	treatment	10460804	8377658 (80.09%)	1135375 (10.85%)	403678 (3.86%)	544093 (5.20%)	60.10
25-E6CK1	E6	control	10467860	8384231 (80.09%)	1138436 (10.88%)	410467 (3.92%)	534726 (5.11%)	60.90
26-E6CK2	E6	control	12215884	10118474 (82.83%)	1208141 (9.89%)	416282 (3.41%)	472987 (3.87%)	63.40
27-E6CK4	E6	control	11095134	8994149 (81.06%)	1162509 (10.48%)	409658 (3.69%)	528818 (4.77%)	64.90
28-E6T3	E6	treatment	13788707	11371696 (82.47%)	1268826 (9.20%)	532741 (3.86%)	615444 (4.46%)	65.40
29-E6T4	E6	treatment	10925285	8899226 (81.46%)	1118063 (10.23%)	405697 (3.71%)	502299 (4.60%)	60.70
30-E6T6	E6	treatment	9292220	7400778 (79.64%)	1129840 (12.16%)	316226 (3.40%)	445376 (4.79%)	61.10
31-E7CK1	E7	control	9392769	7734925 (82.35%)	935358 (9.96%)	330457 (3.52%)	392029 (4.17%)	62.70
32-E7CK2	E7	control	12251141	9774171 (79.78%)	1509440 (12.32%)	403030 (3.29%)	564500 (4.61%)	62.80
33-E7CK3	E7	control	11971446	9806526 (81.92%)	1171496 (9.79%)	444872 (3.72%)	548552 (4.58%)	64.30
34-E7T1	E7	treatment	13068308	10818141 (82.78%)	1299056 (9.94%)	439061 (3.36%)	512050 (3.92%)	64.80
35-E7T2	E7	treatment	12283865	9897366 (80.57%)	1324838 (10.79%)	460071 (3.75%)	601590 (4.90%)	61.60
36-E7T3	E7	treatment	9,870,957	7834799 (79.37%)	1184117 (12.00%)	360264 (3.65%)	491777 (4.98%)	61.70

Supplementary Table 2. Function and expression patterns of differentially expressed genes in the six genotypes including PI262090, F487A, E4, E5, E6, and E7 (submitted as Supplementary Dataset due to big size).

Supplementary Table 3. The enriched gene ontology terms of the differentially expressed genes specific to nodulating genotypes.

Term	Term size	List1 percentage	List2 percentage	Adjusted p-value
oxidation-reduction process(GO:0055114)	3139	16.56	7.69	2.30E-05
metabolic process(GO:0008152)	1915	15.92	4.64	9.68E-11
catalytic activity(GO:0003824)	2429	11.78	5.95	3.24E-03
heme binding(GO:0020037)	937	8.28	2.27	4.41E-06
biosynthetic process(GO:0009058)	392	7.01	0.92	6.54E-10
sequence-specific DNA binding transcription factor activity(GO:0003700)	952	6.05	2.32	7.88E-03
response to oxidative stress(GO:0006979)	251	4.46	0.59	2.25E-06
peroxidase activity(GO:0004601)	248	4.14	0.59	1.29E-05
extracellular region(GO:0005576)	115	1.91	0.27	1.17E-02
cell wall macromolecule catabolic process(GO:0016998)	92	1.91	0.21	3.61E-03
regulation of transcription, DNA-templated(GO:0006355)	5	1.59	0	7.94E-09
monosaccharide binding(GO:0048029)	5	1.59	0	7.94E-09
positive regulation of mitotic nuclear division(GO:0045840)	5	1.59	0	7.94E-09
negative regulation of transcription, DNA-templated(GO:0045892)	5	1.59	0	7.94E-09
plant-type cell wall organization(GO:0009664)	61	1.27	0.14	4.24E-02
chitinase activity(GO:0004568)	43	1.27	0.1	1.33E-02
chitin catabolic process(GO:0006032)	43	1.27	0.1	1.33E-02
chitin binding(GO:0008061)	12	1.27	0.02	1.40E-04
phosphorylation(GO:0016310)	47	1.27	0.11	1.74E-02
response to endogenous stimulus(GO:0009719)	4	1.27	0	6.91E-07
response to organic substance(GO:0010033)	4	1.27	0	6.91E-07
cellular process(GO:0009987)	4	1.27	0	6.91E-07
cytokinin metabolic process(GO:0009690)	22	0.96	0.05	2.11E-02
cytokinin dehydrogenase activity(GO:0019139)	22	0.96	0.05	2.11E-02

transcription factor complex(GO:0005667)	21	0.96	0.04	1.90E-02
endopeptidase inhibitor activity(GO:0004866)	19	0.96	0.04	1.49E-02
inositol-3-phosphate synthase activity(GO:0004512)	4	0.96	0	1.40E-04
inositol biosynthetic process(GO:0006021)	4	0.96	0	1.40E-04
phospholipid biosynthetic process(GO:0008654)	21	0.96	0.04	1.90E-02
nicotianamine synthase activity(GO:0030410)	4	0.96	0	1.40E-04
nicotianamine biosynthetic process(GO:0030418)	4	0.96	0	1.40E-04
defense response to other organism(GO:0098542)	3	0.96	0	4.50E-05
cellular response to organic substance(GO:0071310)	3	0.96	0	4.50E-05
single-organism process(GO:0044699)	3	0.96	0	4.50E-05
regulation of cellular process(GO:0050794)	3	0.96	0	4.50E-05
regulation of transcription(GO:0045449)	3	0.96	0	4.50E-05
proline dehydrogenase activity(GO:0004657)	4	0.64	0	1.33E-02
proline catabolic process(GO:0006562)	4	0.64	0	1.33E-02
single-organism metabolic process(GO:0044710)	2	0.64	0	2.69E-03
response to chitin(GO:0010200)	2	0.64	0	2.69E-03
transferase activity, transferring acyl groups other than amino-acyl groups(GO:0016747)	2	0.64	0	2.69E-03
defense response to fungus, incompatible interaction(GO:0009817)	2	0.64	0	2.69E-03
anthranilate N-benzoyltransferase activity(GO:0047672)	2	0.64	0	2.69E-03
cytokinin binding(GO:0044373)	2	0.64	0	2.69E-03
single organismal cell-cell adhesion(GO:0016337)	2	0.64	0	2.69E-03
negative regulation of cytokinin-activated signaling pathway(GO:0080037)	2	0.64	0	2.69E-03
transferase activity, transferring glycosyl groups(GO:0016757)	2	0.64	0	2.69E-03
stomatal complex morphogenesis(GO:0010103)	2	0.64	0	2.69E-03
anther wall tapetum cell fate specification(GO:0010234)	2	0.64	0	2.69E-03
transmembrane receptor protein kinase activity(GO:0019199)	2	0.64	0	2.69E-03
microsporogenesis(GO:0009556)	2	0.64	0	2.69E-03
response to molecule of bacterial origin(GO:0002237)	2	0.64	0	2.69E-03

meiotic nuclear division(GO:0007126)	2	0.64	0	2.69E-03
serine family amino acid metabolic process(GO:0009069)	2	0.64	0	2.69E-03

Note: List 1 corresponds to the DEGs associated with GO terms assigned; List 2 corresponds to the remaining genes in A and B genomes associated with GO terms.

Supplementary Table 4. Peanut genes orthologous to previously identified nodulation related genes in other species (submitted as Supplementary Dataset due to big size).

Supplementary Table 5. Expression levels and primer sequences of selected differentially expressed genes using qRT-PCR.

Gene name	RNA-seq regulation	E6 (treatment vs control)			E7 (treatment vs control)			qRT-PCR consistent with RNA-seq	Forward primer sequence	Reverse primer sequence
		First bio rep $2^{-\Delta\Delta Cq}$	Second bio rep $2^{-\Delta\Delta Cq}$	Average $2^{-\Delta\Delta Cq}$	First bio rep $2^{-\Delta\Delta Cq}$	Second bio rep $2^{-\Delta\Delta Cq}$	Average $2^{-\Delta\Delta Cq}$			
Aradu. 0KB9D	UP	7.600	11.410	9.505	0.820	1.052	0.936	Yes	GGAGCTTTGT CACTAGGCGT	CAGTCCATCGGT GTCTAGTG
Aradu. 398CK	UP	8.666	6.175	7.420	0.417	1.324	0.870	Yes	GGCGTTTGGTT GACAGGAAT	CCGAGAAAGAA TCACGAACTCTG
Aradu. 62DXS	UP	2.214	1.320	1.767	0.892	1.332	1.112	Yes (<2 fold change)	CGTCTTCTTCG GACTCTTCG	CAGGATCCGGTC TTCTTCTG
Aradu. D5TX4	UP	13.590	2.605	8.098	0.288	1.052	0.670	Yes	GACAAGTTTG GACCGGTGAT	ATGTTGGCGTGG TGACAGTA
Aradu. T3S5X	DOWN	0.550	0.614	0.582	2.563	1.932	2.248	Yes (<2 fold change)	GAGGTACCGT TCTGCTCCAA	ACAACCATGTCC CAGAGGAG
Aradu. V08Y1	UP	2.497	3.487	2.992	0.483	0.420	0.452	Yes	GAGGAGGCAC TAGGCAGTGT	CCCCTACTTCTT CCCACCTG
Araip.3 UM1W	UP	6.259	3.733	4.996	1.481	1.945	1.713	Yes	GGCCAAAGAC AGTTGGAAGA	TGCAGCCACTGA GATGGTAG
Araip.9 7GAP	UP	4.052	53.538	28.795	1.580	0.844	1.212	Yes	TCAGATCAAG ACAGCCGTTG	GACCACCTCCCA AAACAGAA
Araip.I	UP	4.548	0.954	2.751	1.843	1.460	1.651	Yes	GTTTCAGGCTT	AGCTTGCGTGAA

G14B									GCACACATC	GGAATCAC
Araip. NL2P7	UP	14.848	21.275	18.062	1.046	0.911	0.979	Yes	GTATTCATGTT CTCCATCACC	GGGCTGGTGTCA AATATGTCA

Supplementary Figure 1. Venn diagrams showing shared gene families among *Arachis hypogaea*, *Lotus japonicus*, and *Medicago truncatula* (OrthoMCL 2.0.7, e-value <1e-05). The three numbers under species names, separated by "/", are the number of gene families, the number of genes in the families, and the total number of genes or protein sequences input into OrthoMCL. A gene family contains at least two genes. (A) The comparison among DEGs specifically in nodulating genotypes and all genes in *Lotus japonicus* and *Medicago truncatula*. (B) The comparison among all genes in these three species.

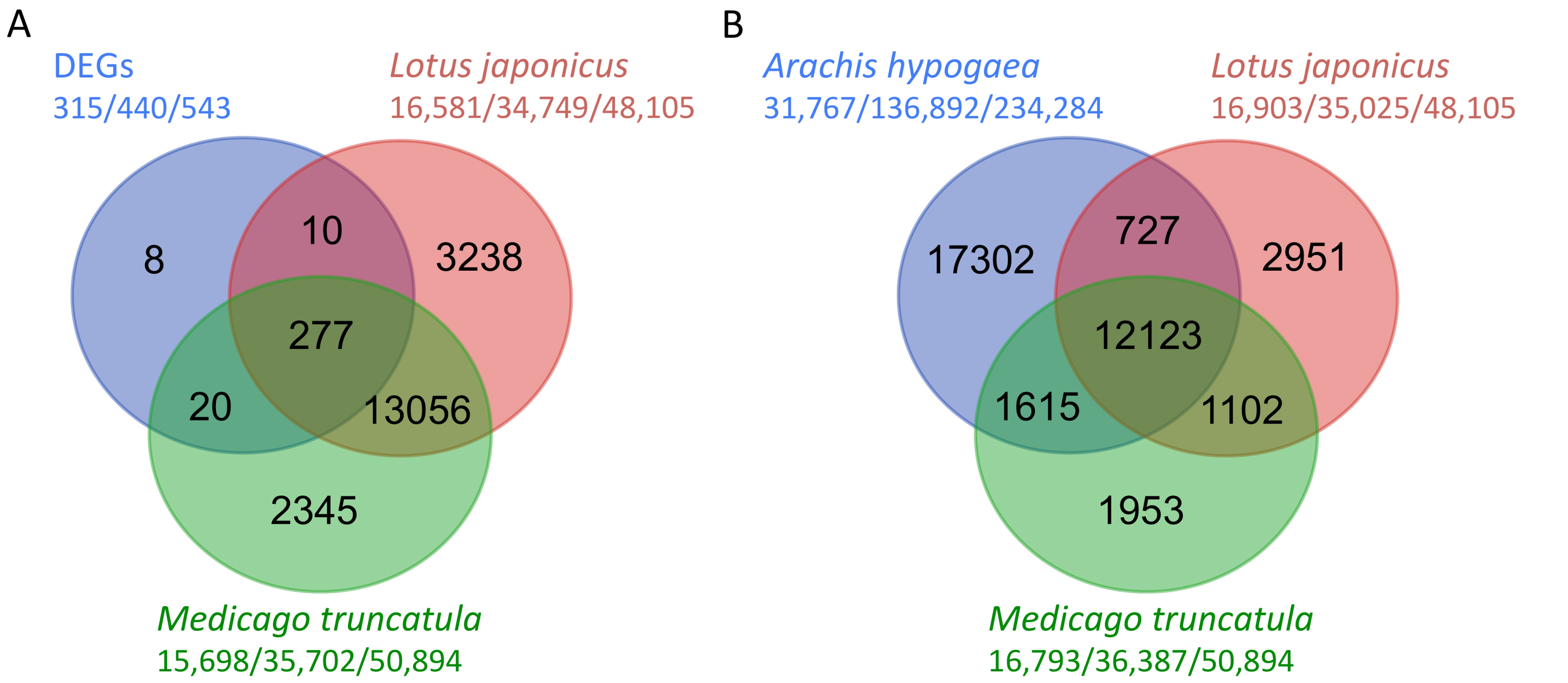
A
DEGs
315/440/543
Lotus japonicus
16,581/34,749/48,105
8
10
3238
277
20
13056
2345
Medicago truncatula
15,698/35,702/50,894
B
Arachis hypogaea
31,767/136,892/234,284
Lotus japonicus
16,903/35,025/48,105
17302
727
2951
12123
1615
1102
1953
Medicago truncatula
16,793/36,387/50,894
